# Beneficial Effects of Alcohol in Old Age

**Published:** 1882-01

**Authors:** F. H. O’Brien

**Affiliations:** Atlanta, Ga.


### BENEFICIAL EFFECTS OF ALCOHOL IN OLD AGE.

By F. H. O'BRIEN, M.D., Atlanta, Ga.

Thinking that the following case may be of some interest, I report it: Was called on the morning of January 12th, 1880, to see Lavinia Cobb, colored, aged one hundred and five years, mother of five children, who has always been remarkable for good health. The day previous she wandered from the home of her great grand-daughter, with whom she was living, and late in the afternoon, when about two miles from the city, fell into a branch and was waist deep in water. Not having sufficient strength to extricate herself, she remained in that position until the next morning, when a party of men on the way to their work discovered her, and placing her in a wagon brought her to her home, where I saw her a few moments after her arrival. Her condition was critical in the extreme; she was suffering with a severe chill; was insensible and almost pulseless. I ordered the wet clothing to be be removed; had her wrapped in warm blankets and bottles of warm water applied to her extremities. The temperature of the room was elevated by a large fire, and as soon as she was able to swallow, which was about half an hour after her arrival, she was given half an ounce of brandy with an ounce of milk which was repeated every hour during the remainder of the day.

She talked incoherently for awhile, but soon dropped into a refreshing sleep of four hours duration, and on awaking remembered falling into a branch, trying hard to get out and suffering very much with cold. She complained of pain over her left eye and said her left foot was very sore. There was a contusion over the left eye, and on examination, her foot was found to be minus about one-half of the cuticle of the planter surface. She slept nearly all night and the next morning said she felt quite well, with the exception of a slight pain in her chest, which became very severe towards noon and was accompanied by a troublesome cough. A mustard plaster was applied over the chest and stimulants with milk continued at intervals of two hours.

I felt sure that bronchitis was developing and would soon bring the case to a fatal termination, but to my surprise the pain in the chest and the cough disappeared rapidly, and in a few days she was enjoying her usual health, which, as before stated, was remarkable for a person of her age. Alcoholic stimulants, from time immemorial, have been recommended for many of the troubles of the aged, and I think this case strikingly illustrates the efficacy of the remedy.

